# Highly Loaded Fe-MCM-41 Materials: Synthesis and Reducibility Studies

**DOI:** 10.3390/ma2042337

**Published:** 2009-12-15

**Authors:** Malose P. Mokhonoana, Neil J. Coville

**Affiliations:** 1 Department of Chemistry, University of Limpopo, P/Bag X1106, Sovenga, 0727, Polokwane, South Africa; E-Mail: malosem@ul.ac.za (M.P.M.); 2 Molecular Sciences Institute, School of Chemistry, University of the Witwatersrand, P/Bag 3, WITS 2050, Johannesburg, South Africa

**Keywords:** Fe-MCM-41, secondary synthesis, temperature programmed reduction, wustite (FeO), incipient wetness impregnation

## Abstract

Fe-MCM-41 materials were prepared by different methods. The Fe was both incorporated into the structure and formed crystallites attached to the silica. High Fe content MCM-41 (~16 wt%) with retention of mesoporosity and long-range order was achieved by a range of new synthetic methodologies: (i) by delaying the addition of Fe^3+^(aq) to the stirred synthesis gel by 2 h, (ii) by addition of Fe^3+^ precursor as a freshly-precipitated aqueous slurry, (iii) by exploiting a secondary synthesis with Si-MCM-41 as SiO_2_ source. For comparative purposes the MCM-41 was also prepared by incipient wetness impregnation (IWI). Although all these synthesis methods preserved mesoporosity and long-range order of the SiO_2_ matrix, the hydrothermally-fabricated Fe materials prepared via the secondary synthesis route has the most useful properties for exploitation as a catalyst, in terms of hydrothermal stability of the resulting support. Temperature-programmed reduction (TPR) studies revealed a three-peak reduction pattern for this material instead of the commonly observed two-peak reduction pattern. The three peaks showed variable intensity that related to the presence of two components: crystalline Fe_2_O_3_ and Fe embedded in the SiO_2_ matrix (on the basis of ESR studies). The role of secondary synthesis of Si-MCM-41 on the iron reducibility was also demonstrated in IWI of *sec*-Si-MCM-41.

## 1. Introduction

The substitution of various transition elements into open-framework microporous and mesoporous materials [1,2,3,4,5] has received considerable attention because of the need to develop more efficient and stable materials for applications in catalysis, separations, coatings and chemical sensing. In particular, iron-containing zeolites [6] and related molecular sieves are of particular interest because of their unique catalytic activity in various selective gas-phase reactions such as hydrocarbon oxidation [7,8,9], N_2_O decomposition [10,11], synthesis of carbon nanotubes [12,13] and selective catalytic NO and N_2_O reduction in the presence of hydrocarbons or ammonia [14,15,16]. 

Another area of catalysis in which Fe constitutes a key catalyst component is the Fischer-Tropsch synthesis (FTS) [17,18,19]. In this process remote and abundant natural gas as well as renewable biomass sources are converted into high-quality fuels and valuable raw chemicals via synthesis gas [20]. Compared to other metal catalysts for Fischer-Tropsch (FT) synthesis, the iron-based catalyst is distinguished by higher conversion, good selectivity to the lower olefins, and flexibility to the process parameters [21,22]. One of the driving forces behind the rekindled interest in the FTS is the uncertainty in petroleum reserves and the ever-escalating price of crude oil. In the past decade, the use of mesoporous silica (MCM-41 and SBA-15) as a Fischer-Tropsch catalyst support has witnessed a tremendous growth. This has led to Co and Fe catalyst systems with improved catalytic activity and C_5+_ hydrocarbon selectivity, and the absence of mass-transfer limitations in the reaction [20,23,24,25]. These studies also demonstrated that the larger pore size enhanced the selectivity to C_5+_ products in the Fischer-Tropsch reaction on cobalt catalysts supported on periodic mesoporous silicas (SBA-15 and MCM-41) [26].

The activity of the mesoporous silica-supported catalysts for the FTS was found to be affected by the reducibility of cobalt and iron oxides. This dependence of catalytic activity and selectivity on the reducibility of the metal species was also observed on non-silica supports in other reactions [27,28,29,30,31]. Since the catalyst reducibility is such an important feature of FTS catalysts, one of the aims of this paper is to study the reducibility of differently-prepared Fe-MCM-41 materials. To date, no previous study has been dedicated to the investigation of the reducibility of mesoporous silica-supported FTS-type catalysts as a function of preparation method.

The synthesis of iron-containing MCM-41 is, however, not without problems. Fe-MCM-41 was hydrothermally synthesized for the first time in 1995 by Yuan *et al.* [32]. This was followed by the room temperature synthesis of Fe-containing hexagonal mesoporous silica (HMS) molecular sieve materials in subsequent years, using neutral hexadecylamine as a surfactant molecule [33,34]. In addition to these direct synthesis and conventional impregnation methods, Fe-MCM-41 has also been prepared by template ion exchange [11,35]. It was not until the work of Samanta *et al.* [36] that iron-rich and highly ordered Fe-MCM-41 containing predominantly tetrahedrally-coordinated Fe^3+^ in the silica network was prepared under mild alkaline (pH = 8–8.5) hydrothermal conditions. The optimum limit of iron loading for the resulting ordered mesophase was 8.2 wt%, beyond which a disordered iron oxide/silica phase was observed [36]. This finding suggests that typical FTS Fe loadings cannot be achieved by direct synthesis without compromising the structure and long-range order of MCM-41. This current study sought to evaluate the metal content limitation in the one-pot synthesis of Fe-containing MCM-41 materials by investigating methods of Fe incorporation prior to hydrothermal treatment, with the Fe loading restricted to 16 wt%. This work has thus focused on the preparation of mesoporous ferrisilicate and Fe_2_O_3_/silica materials using incipient wetness impregnation and different versions of the one-pot synthesis. Temperature-programmed reduction (TPR) was employed as a probe chemical reaction to examine the reducibility of the resulting ferrisilicates. In evaluating the secondary synthesis of Si-MCM-41, a three-step TPR profile was observed. This may either be due to a wustite (FeO) intermediate or the reduction of Fe species in framework and extra-framework silica environments.

## 2. Experimental

The synthesis of Si-MCM-41 was carried out following the delayed neutralization approach reported by Lin *et al.* [37], using sodium silicate (Merck: 25.5–28.5% SiO_2_, 7.5–8.5% Na_2_O) as SiO_2_ source and cetyltrimethylammonium bromide (CTAB) (Aldrich: 98%) as template. Unless otherwise stated, all syntheses were carried out using water glass (sodium silicate) as SiO_2_ source. The metal-containing MCM-41 derivatives were synthesized by inclusion of Fe(NO_3_)_3_.9H_2_O. The synthesis gel pH was adjusted to 10 using dilute HNO_3_ solution. For phase identification in XRD and TPR experiments, bulk Fe_2_O_3_ (Merck: 99%) precalcined at 560 °C for 6 h was used as reference.

### 2.1. Synthesis Procedures

The synthesis of the Fe-containing MCM-41 was carried out in a similar way to that used to prepare siliceous MCM-41, at both ambient temperature and under hydrothermal conditions for various lengths of time. The Fe metal precursor was either added in a one-pot synthesis to a silica source (T = 100 °C, t = 2 days), or in a post-synthesis addition (indirect synthesis) to the calcined pre-formed Si-MCM-41 by incipient wetness impregnation. Depending on the amount of metal precursor used during the one-pot synthesis, the pH adjustment to 10 also required the addition of a base. The one-pot synthesis of Fe-MCM-41 was extended by investigating the use of milder synthesis conditions, with the synthesis temperature being lowered to 80 °C and the crystallization time reduced to 6 hours, similar to conditions used by Thitsartarn *et al.* [38]. This milder synthesis was also carried out under magnetic stirring in polypropylene bottles. Syntheses of Fe-MCM-41 were performed using calcined Si-MCM-41 as a silica source for the one-pot hydrothermal synthesis at 100 °C. No elemental analysis was done on the final materials and therefore, the quoted metal loadings are nominal. The principal assumptions made in the quantification of metal contents were that hydrolysis of the silicate is complete and that there is no loss of Fe during (i) solution transfer, (ii) the washing step after filtration, and (iii) calcination.

#### 2.1.1. Direct Synthesis: (Aqueous) Acid-Mediated Route

In a modified procedure for the metal incorporation into the synthesis gel, the metal precursor was dissolved in water or in a dilute (1 M) acid solution and then added to the highly alkaline water-glass/CTAB/H_2_O mixture at room temperature. After homogenizing by magnetic stirring for 1 h and adjusting the pH to 10, the synthesis mixture was subjected to either room temperature or hydrothermal synthesis. After crystallization, the solid product was recovered by filtration, washed copiously with distilled water until a negative Br^-^ test was achieved, dried at ambient and then calcined at 560 °C for 6 h.

#### 2.1.2. Direct Synthesis: The Hydroxide Precipitate Route

In this method, an aqueous solution of Fe(III) was precipitated with a stoichiometric amount of an alkaline solution and the resulting gelatinous metal hydroxide (in its mother liquor) was added to an aqueous CTAB solution either before or after the silica source addition. After synthesis (room temperature or 100 °C), the solid was again recovered by filtration, washed free of Br^-^ ions and then calcined at 560 °C for 6 h.

#### 2.1.3. Post-synthesis Metal Incorporation: Incipient Wetness Impregnation (IWI)

The Fe(III) precursor was dissolved in a volume of a solvent (1 M HNO_3_ or distilled water) that is just sufficient to fill the pores of the support. After solution addition the resulting material was allowed to dry at room temperature and then overnight in an oven maintained at 110 °C, followed by calcination at 560 °C for 6 h or 450 °C for 12 h.

All the solid products obtained following the procedures detailed above, except those obtained by incipient wetness impregnation, were recovered by filtration, washed free of Br^-^ ions, dried at room temperature and then calcined at 500–560 °C for 6–12 h.

### 2.2. Characterization of Fe-MCM-41

The transition metal-containing mesoporous derivatives of MCM-41 were characterized by X-ray diffraction (XRD), Brunauer-Emmet-Teller (BET) surface area measurement, high resolution transmission electron microscopy (HRTEM), thermogravimetric analysis (TGA), temperature programmed reduction (TPR) and electron spin resonance (ESR) spectroscopy. In order to identify the Fe phases formed in the mesopores of MCM-41 upon calcination, bulk Fe_2_O_3_ (Merck) was used as a standard and the XRD pattern of the bulk phase was compared with those of the metal-containing MCM-41.

The XRD patterns of these materials were recorded on a Philips PW 1710 Automated Powder Diffractometer using a graphite monochromator-filtered Cu K_α_ radiation (λ = 0.15406 nm), at a generator tension of 40 kV and generator current of 20 mA. A TGA/SDTA 851e (Mettler Toledo) equipped with a TSO 801 RO sample robot was used to record the TGA curves. The analysis was done in the temperature range 25–900 °C at a heating rate of 5.00 °C/min, with an air flow rate of 20.0 mL/min and synchronization was enabled. Surface area measurements were performed on a Micromeritics ASAP 2010 surface analyzer by adsorbing gaseous N_2_ at liquid nitrogen temperature. The samples (0.2–0.3 g) were degassed at 300 °C until a vacuum pressure of 2–4 μm Hg was obtained, prior to analysis. A relative pressure range (P/P_o_) of 0.05–0.25 was used in the analysis. The microstructure of MCM-41 derivatives was investigated using a JEOL 2010 high resolution transmission electron microscope (HRTEM), using an accelerating voltage of 200 kV. In the sample preparation, these materials were ground to a thin paste in ethyl alcohol, mounted on a carbon-coated copper grid by dipping the grid in the paste, air-dried at ambient temperature and then loaded into the microscope chamber for analysis. The X-band (a cavity operating at 9 GHz) ESR spectra of the metal-containing MCM-41 complexes were recorded at room temperature using a Bruker ESP 380 (Pulse and Continuous Wave) spectrometer, using a field modulation of 100 kHz, an amplitude modulation of 5 G and a microwave power of 2.2 mW.

The reducibility of the Fe-containing MCM-41 derivatives was investigated on a home-built TPR set-up. The reactor used was a U-shaped quartz tube and the sample was held in position by quartz wool plugs. Prior to the TPR experiment, the reactor and its contents were flushed with helium gas using a flow rate of 30 mL/min under controlled heating to 150 °C, and held isothermal for 30 minutes. Then the inert gas was switched to a 5% H_2_/He mixture and the reduction was performed at a controlled heating rate of 7.5 °C/min from ambient to 800 °C. (A cell containing oxysorb was used to remove water formed during the reduction). The hydrogen consumption was monitored by a thermal conductivity detector (TCD).

## 3. Results and Discussion

In this study, two versions of the one-pot synthesis of Fe-MCM-41 were used. One version involved the addition of an acidified aqueous solution (1 M HNO_3_) of Fe(III) to the synthesis gel prior to hydrothermal treatment, while the other involved prior precipitation of the Fe(III) precursor with a base before addition of the resulting slurry to the synthesis gel and subsequent hydrothermal treatment. In addition, IWI was also used to prepare Fe-MCM-41. The characterization results are discussed according to the synthesis method used.

### 3.1. Acidified Aqueous Incorporation of Iron in MCM-41

**Figure 1:**
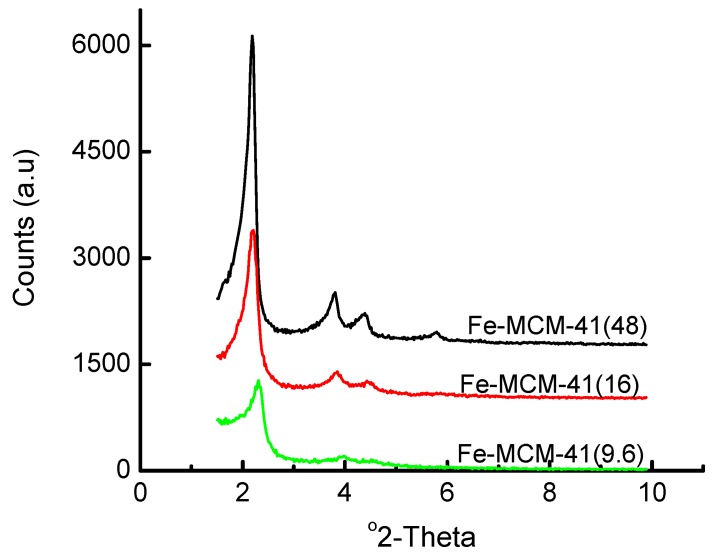
Variation of the XRD patterns of Fe-MCM-41 with metal content for the 100 °C synthesis in which the Fe(III) precursor was dissolved in acidic aqueous solution before adding to the synthesis mixture. The Si/Fe mole ratio is given in brackets.

The idea of using an acid solution for introducing the Fe(III) was to minimize local precipitation of Fe oxides and hydroxides in the alkaline water glass-derived synthesis gels. Figure 1 shows the XRD patterns of Fe-containing MCM-41 mesoporous materials prepared by adding a 1 M HNO_3_ solution of ferric nitrate to the stirred water glass/CTAB/H_2_O synthesis mixture. It is apparent from this figure that the mesoporous long-range order decreases with the increasing amount of Fe used in the synthesis gel, signalled by the decrease in intensity and eventual loss of the higher order peaks [(110) and (200)] in the XRD patterns. This trend in structural variation as a function of the Fe content is further supported by the data in Table 1, and suggests 8.8 wt% to be the optimum Fe loading, similar to that reported by Samanta *et al.* [36].

It is important to note that no (100) Bragg peak was detected when the synthesis gel Fe content was increased to 16 wt%, further confirming the breakdown of the mesoporous structure at Fe loadings beyond 8.8 wt%. Regardless of this apparent loss of mesoporosity at 16 wt% Fe content, the material still possessed a high surface area (522.2 m^2^/g). *Figure SI 1* (Supplementary Material) illustrates these observations by showing a rapid decrease in lattice parameter as the Fe loading increases beyond 2 wt%. Since this decrease is associated with mesostructural collapse, it can be deduced from this figure that the optimal amount of Fe that can be incorporated into the mesoporous silica matrix with maximal retention of long-range order is below 2 wt%. The BET data agree with the XRD results as shown in *Figure SI 1B*. All the measured BET areas are high (800–950 m^2^/g) and characteristic of mesoporous materials. 

**Table 1:** Variation of the lattice parameter of Fe-MCM-41 with the synthesis gel Fe content.

Fe precursor mass/g	Gel Si/Fe ratio	Wt% Fe	a_o_/Å
1.00	48	~2	46.6
3.00	16	~5	46.4
5.00	9.6	~8.8	44.1
10.00	4.8	~16	(100) not detected*

* The lattice parameter, a_o_, is obtained from the (100) XRD peak.

**Figure 2:**
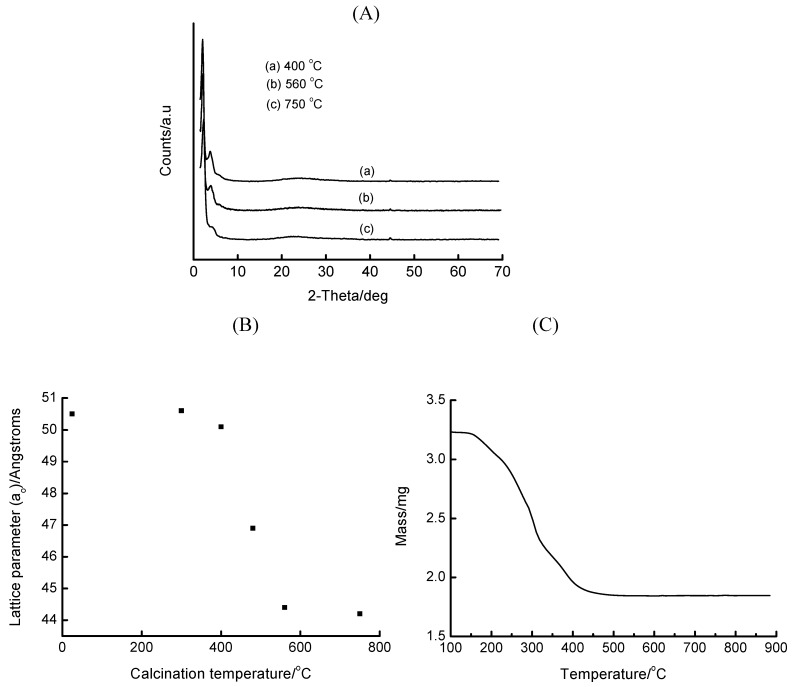
The thermal behaviour of 5 wt% Fe-MCM-41 prepared hydrothermally by the HNO_3_ route at 100 °C for 2 days: the XRD patterns (A), the lattice parameter (B) and TGA plot (C).

Studies with the 5 wt% Fe-MCM-41 (Si/Fe = 16) synthesized through this route reveal interesting features as depicted in Figure 2. The XRD patterns show that this material undergoes a reduction in mesostructure long-range order as the calcination temperature increases from 400 to 750 °C, as marked by the gradual disappearance of higher order peaks. Interestingly, the loss of surfactant at various calcination temperatures is accompanied by a contraction in the unit cell parameter (a_o_). The corresponding TGA plot confirms this finding. Both these plots suggest that the surfactant is completely removed at temperatures *ca*. 500 °C.

**Figure 3:**
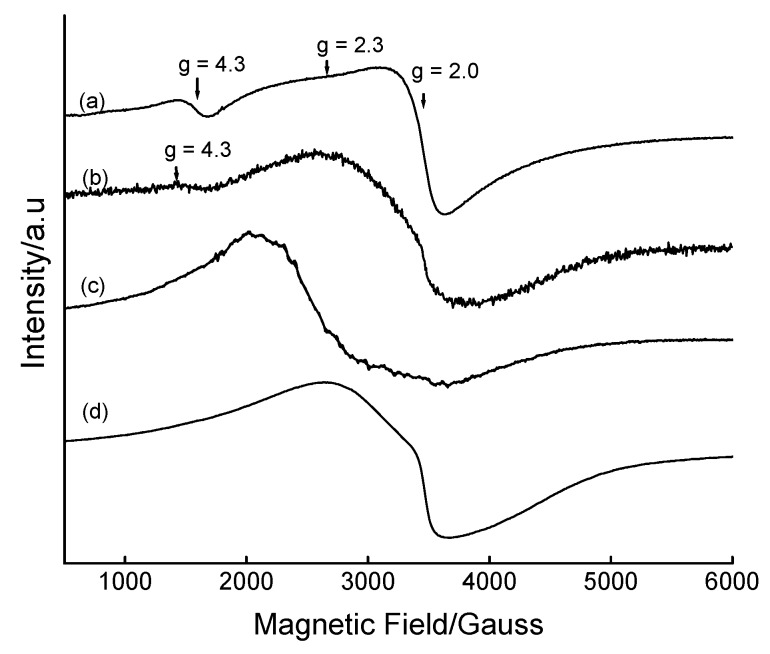
Room temperature X-band ESR spectra of 5 wt% Fe-MCM-41 prepared at 100 °C for 2 days as a function of calcination temperature: (a) as-synthesized, (b) 300 °C for 6 h, (c) 400 °C for 6 h, and (d) 560 °C for 6 h.

The coordination environment of the Fe(III) in the 5 wt% Fe-derivatized mesoporous silica matrix as a function of calcination was elucidated by room temperature ESR spectroscopy (Figure 3). The as-synthesized material [Figure 3 (a)] shows the existence of at least three signals corresponding to different environments, characterized by different values of the Lande *g*-factor. The dominant iron environments are represented by ESR signals at *g* = 4.3 and *g* = 2.0, which are respectively assigned to Fe^3+^ in tetrahedral framework sites and in octahedral (cation exchange or interstitial) sites [38,39]. Similar observations have also been reported on other iron-containing MCM-41 materials [32,40]. Upon calcination at 300 °C for 6 h, the signal at *g* = 4.3 decreased in intensity and disappeared at higher calcination temperatures, suggesting the ejection of Fe^3+^ from framework sites during calcination. The increased linewidth of the *g* = 2.0 signal with calcination (∆_pp_ = 555, 1241 and 1624 G for calcination temperatures of 25, 300 and 400 °C, respectively) may suggest the migration of Fe^3+^ ions from framework to extraframework sites. Only a single signal with *g* ≈ 2.0 is observed in materials that have been calcined at temperatures above 300 °C, confirming the presence of Fe in one type of coordination environment. The symmetry and size of this peak also shows some dependence on the calcination temperature. The breadth and higher g value for the material calcined at 400 °C (*g* ≈ 2.4) may suggest the presence of iron oxyhydroxides [39]. The dislodgement of iron to extraframework positions as a result of calcination has also been reported by Perez-Ramirez *et al.* [41] on the basis of techniques other than ESR. In this study, no analysis was performed to quantify the amount of Fe in each type of coordination site.

**Figure 4:**
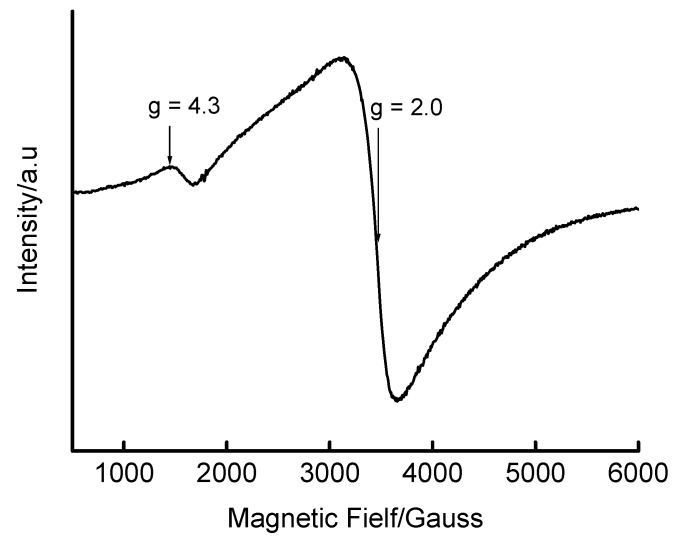
Room temperature X-band ESR spectrum of as-synthesized 8.8 wt% Fe-MCM-41 prepared by room temperature synthesis for 5 days with a 1 M HNO_3_ solution of Fe(III).

Upon increasing the iron content in Fe-MCM-41 to 8.8 wt% (prepared at room temperature for 5 days), the ESR spectrum in Figure 4 was obtained. The relative peak sizes in this figure suggest there is relatively less Fe in the framework (*g* ≈ 4.3) compared to extraframework Fe species (*g* ≈ 2.0).

**Figure 5:**
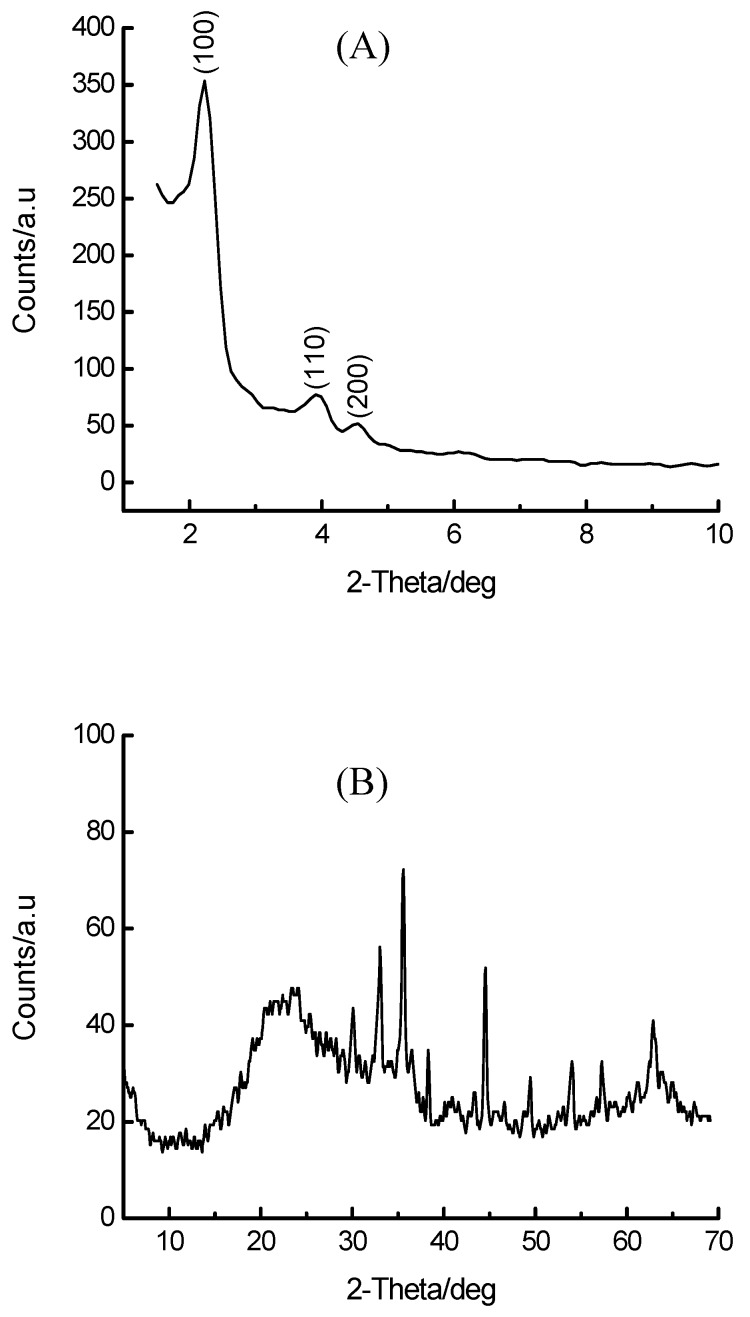
The low-angle (A) and high-angle (B) components of the XRD pattern of the 16 wt% Fe-MCM-41 material synthesized hydrothermally using calcined Si-MCM-41 as a SiO_2_ source.

In order to address the structural breakdown accompanying the one-pot synthesis of 16 wt% Fe-MCM-41, water glass was replaced by calcined Si-MCM-41 as a SiO_2_ source and the resulting synthesis mixture (pH 10) was subjected to hydrothermal treatment at 100 °C for 2 days. The use of Si-MCM-41 as a SiO_2_ source for the secondary synthesis of *sec*-Si-MCM-41 was previously reported by Mokaya [42] and was found to enhance the hydrothermal stability of this material by thickening and recrystallizing the amorphous pore walls of primary Si-MCM-41. The benefits of this method when applied to Fe-MCM-41 synthesis are shown in Figure 5, which shows both the low-angle and the high-angle region of the XRD pattern of the resulting material after calcination. Both mesoporosity and long-range order of the silica matrix are unambiguously confirmed by the appearance of the first order peak (100) as well as well-resolved higher order peaks (110) and (200). The high-angle region shows a characteristic pattern reminiscent of Fe_2_O_3_. The full XRD pattern is shown in *Figure SI 2*. This XRD pattern shows, in addition, the existence of an amorphous material, as witnessed by the broad feature centred at around 20–25 ^o^2θ. In addition, intense metal oxide peaks are observed in the region beyond 30 ^o^2θ. These peaks account for the observed TPR behaviour of this material (see later). It is striking to note that a similar synthesis using water glass as SiO_2_ source led to the destruction of mesoporosity and long-range order. The novelty of this method can also be realized by considering the lattice parameters of 1^o^ Si-MCM-41, 2^o^ Si-MCM-41 and 16 wt% Fe-*sec*-Si-MCM-41 prepared at 100 °C for 2 days, which are 47.1, 46.1 and 44.8 Å, respectively. The decrease in a_o_ upon secondary synthesis has been attributed to thickening of the pore walls [42], leading to decreased pore sizes. On the other hand, the 5 wt% Fe-MCM-41 material prepared by IWI on 2^o^ Si-MCM-41 showed retention of the structural integrity with a_o_ = 46.7 Å compared to 46.1 Å in the support material.

High resolution transmission electron microscopy also confirmed retention of mesoporous long-range order when low Fe contents are used (see *Figure SI 3*). This observation is in agreement with that given by Thitsartarn *et al.* [38], who also identified the optimal synthesis conditions for maintaining the hexagonal array of the MCM-41 structure as 60 °C for 7 h followed by calcination at 550 °C. The important outcome of this study is that as the Fe content is increased, the Fe species is not in the structure after 2 wt% loading. Also, Fe species are not in the structure at all when Fe is introduced post-synthetically.

### 3.2. Base-Mediated Incorporation of Iron in MCM-41

Since the aqueous acid-mediated synthesis route afforded only low metal loadings (~9 wt%), an alternative approach was to add the Fe precursor as a gelatinous hydroxide precipitate (slurry) to the synthesis gel prior to hydrothermal or room temperature synthesis.

**Figure 6:**
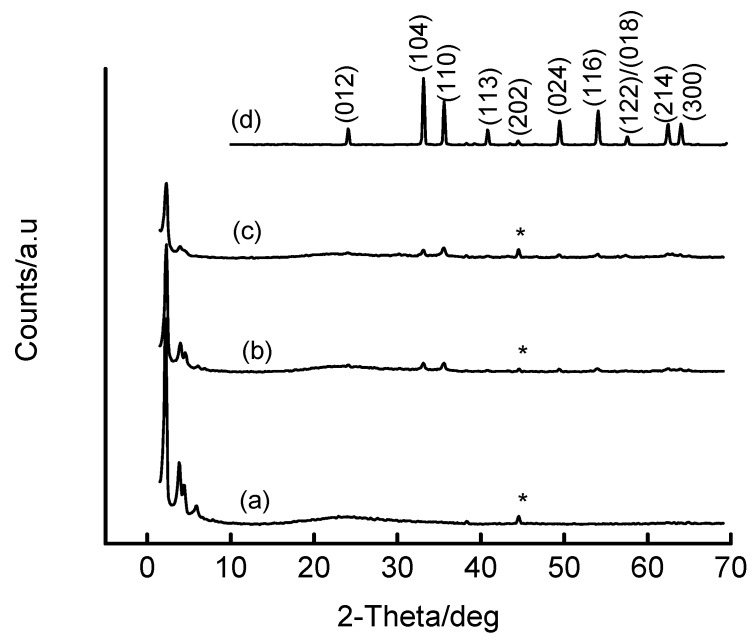
XRD patterns of Fe-MCM-41 prepared hydrothermally (100 °C, 2 days) via the OH^-^ route and calcined at 560 °C for 6 h: (a) 5 wt% Fe-MCM-41, (b) 10 wt% Fe-MCM-41, (c) 16 wt% Fe-MCM-41 and (d) bulk Fe_2_O_3_. The peak marked * is a contribution from the Al sample holder.

**Figure 7:**
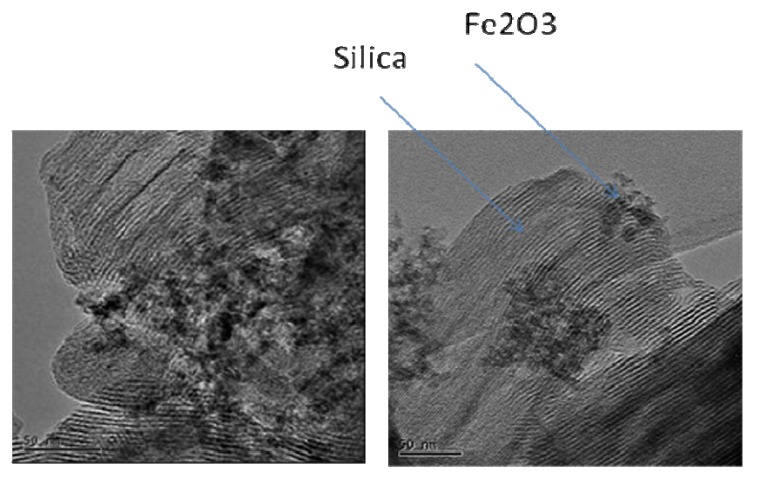
HRTEM micrographs of two different regions of 5 wt% Fe-MCM-41 prepared via the OH^-^ route at 100 °C for 2 days, and calcined at 560 °C.

Figure 6 shows the effect of Fe content on the structural features of Fe-MCM-41 when a freshly-precipitated Fe(OH)_3_ slurry is used as the source of Fe. Interestingly, all Fe-MCM-41 materials produced by this method possess long-range order in their pore systems, evidenced by the presence of higher-order peaks in addition to the prominent (100) peak. Also, metal oxide peaks become increasingly apparent when the Fe loading exceeds 5 wt% Fe, and these peaks are characteristic of Fe_2_O_3_. The Fe species is well dispersed in the resulting ferrisilicate when low Fe loadings are used. In contrast Fe addition by this method preserves the mesostructure in the final material even at loadings of up to 20 wt% (see Table SI 1). The HRTEM micrograph of the 5 wt% Fe-MCM-41 material prepared through this approach is shown in Figure 7. The long-range order of the channel system in the mesoporous silica support is readily apparent. In addition, the iron oxide clusters are clearly visible as a separate phase agglomerated outside the channel structure of the silicate phase. At this stage, no analysis has been done to establish the Fe content inside the porous structure. Data from the BET analysis of these materials also parallels the XRD information as shown in Table SI 1. The general trend is a decrease in lattice parameter (a_o_) and BET surface area as the Fe content increases, which can be attributed to increasing structural destruction and pore blockages of the support at high metal loadings.

The nature of the precipitating agent has an influence on the structural and textural properties of the 16 wt% Fe-MCM-41 as shown by Figure SI4 and Table 2. Retention of the mesostructure of MCM-41, together with its long-range order of hexagonal channels is clearly demonstrated by the XRD pattern (*Figure SI 4*). The Fe_2_O_3_ peaks in the high-angle region of the XRD patterns are more intense for NaOH and triethanolamine (TEA) than for Na_2_CO_3_, and may account for the different reduction profiles observed (see TPR section).

The a_o_ values of the materials in Table 2 suggest that the materials are all mesoporous, although the TMAOH- and TEA-derived samples showed slightly higher values than the alkali metal base-derived materials. The origin of this difference is not clearly understood at the moment, but it can be associated with the higher basicity of the sodium-containing bases, which may induce partial solubility of the framework silicate. Notably, this preparation method affords materials of reasonably high surface area. Also, the use of alkali-free bases results in higher S_BET_ values than observed for the alkali metal containing base (NaOH).

**Table 2:** Variation of the lattice parameter (a_o_) of 16 wt% Fe-MCM-41 with the identity of he precipitant for Fe(III).

Base	a_o_/Å	S_BET_/m^2^. g^-1^
Na_2_CO_3_	44.9	-
NaOH	44.4	546
N_2_H_4_.H_2_O	46.1	617
TMAOH*	51.0	591
(HOCH_2_CH_2_)_3_N	42.0	-

* TMAOH = tetramethylammonium hydroxide, (CH_3_)_4_NOH.

### 3.3. Incipient Wetness Impregnation (IWI) of Si-MCM-41 with Fe(III) Solutions

**Figure 8:**
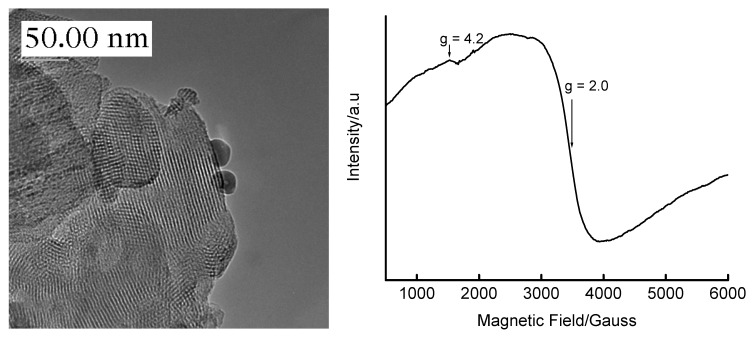
HRTEM micrograph (*left*) and ESR spectrum (*right*) of 16 wt% Fe-MCM-41 prepared by the IWI method using a HNO_3_ acid solution of Fe(III), dried overnight at 110 °C and calcined at 560 °C for 6 h.

One of the advantages of the IWI method is that high metal loadings can be attained without any significant effect on the structure of the support, *i.e.,* the synthesis starts with an already ordered Si-MCM-41. A plot of a_o_ as a function of Fe content for materials prepared by IWI is shown in *Figure SI 5*. There is very little structural destruction when Fe is introduced by this method. However, the presence of high-angle peaks in the XRD patterns of materials prepared by this method suggests the agglomeration of Fe_2_O_3_ outside the pore channels of the support during calcination (*see* *Figure SI 6*). It is important to note that the mesoporous structure and the long-range order of Si-MCM-41 is preserved up to a loading of 50 wt% Fe, as marked by the persistence of low-angle XRD peaks of these materials. The HRTEM micrograph of the 16 wt% Fe-MCM-41 prepared by IWI (Figure 8) supports retention of mesoporosity and long-range-order of the support, as well as the presence of agglomerated Fe species on the surface. The ESR spectrum (Figure 8) of this material confirms the minimal incorporation of Fe in tetrahedral sites in the calcined material.

**Figure 9:**
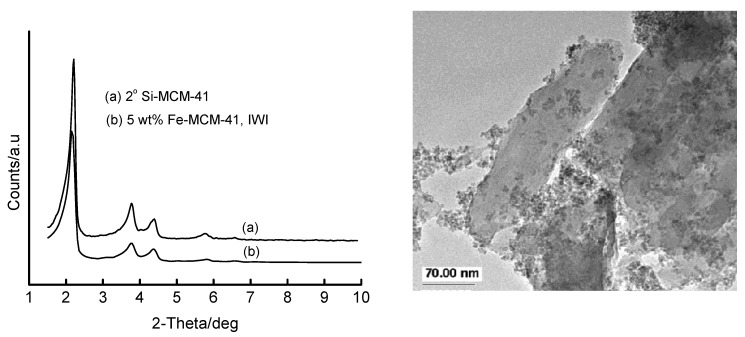
XRD patterns (*left*) and HRTEM micrograph (*right*) of 5 wt% Fe-MCM-41 prepared by IWI with aqueous Fe(III) on *sec*-Si-MCM-41.

The use of secondary Si-MCM-41, *i.e.,* the Si-MCM-41 synthesized using primary Si-MCM-41 as a silica source in the secondary synthesis of the material, as support during the IWI preparation of 5 wt% Fe-MCM-41 showed interesting features as depicted in Figure 9. The *sec*-Si-MCM-41 is itself highly ordered as shown by intense higher-order XRD peaks. These peaks are least affected by the incorporation of the iron species because of the enhanced quality of the support by secondary synthesis. 

On the other hand, HRTEM analysis showed that Fe oxides are highly dispersed outside the channel structure of *sec*-Si-MCM-41 as a separate phase, and therefore the high-angle XRD pattern (*not shown*) should show the presence of metal oxide peaks. Another noteworthy feature from Figure 9 is the restructured morphology (*i.e.,* the honeycomb structure has changed to larger, elongated and sheet-like particles with thickened walls) of the silica support [42].

### 3.4. Temperature-Programmed Reduction (TPR) Studies

**Figure 10:**
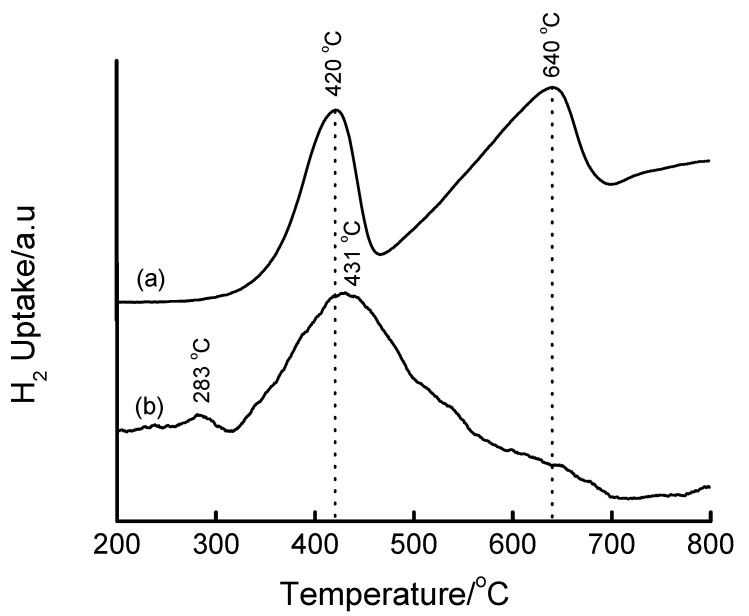
TPR profiles of (a) bulk Fe_2_O_3_ and (b) 8.8 wt% Fe-MCM-41 prepared by HNO_3_-mediated incorporation of Fe(III) at 100 °C for 2 days and calcined at 560 °C for 6 h.

The reducibility of Fe particles in mesoporous silica matrices was evaluated by means of TPR measurements. In the literature [43], the TPR profile of Fe_2_O_3_ showed two peaks at around 400 and 600 °C which are attributed to the reduction of Fe_2_O_3_ to Fe_3_O_4_ followed by Fe_3_O_4_ to Fe, respectively. In this study, the TPR profile of bulk Fe_2_O_3_ (Merck) was recorded, and showed two reduction peaks at 420 and 640 °C (Figure 10(a)), in excellent agreement with the literature [43]. In other cases, up to three reduction peaks have been observed in the TPR profiles of silica-supported Fe catalysts prepared by impregnation and microemulsion methods [44]. These three reduction peaks have been assigned to the reduction steps (i) Fe_2_O_3_ → Fe_3_O_4_, (ii) Fe_3_O_4_ → FeO and (iii) FeO → Fe. Such an assignment of the three reduction peaks was also reported by Chen and Yan [45] on non-silica supports.

Figure 10(b) shows the TPR profile of 8.8 wt% Fe-MCM-41 synthesized by the acidified aqueous solution route at 100 °C for 2 days, and then calcined at 560 °C for 6 h. The profile shows a small peak at ~283 °C and a large one centred at ~431 °C. By analogy with Fe_2_O_3_, the peak at ~431 °C can be assigned to the reduction of Fe_2_O_3_ to Fe_3_O_4_ and the 11 °C shift to higher reduction temperatures relative to Fe_2_O_3_ can be attributed to stabilization of Fe(III) by the silica matrix. The small peak at ~283 °C can be attributed to some highly dispersed Fe phase on the support. The shoulders to the right hand side of the 431 °C peak correspond to a small degree of reduction to metallic Fe, suggesting that the majority of Fe(III) is in the MCM-41 structure. 

In the quest to produce a highly loaded Fe-MCM-41 by direct hydrothermal synthesis, the addition of a water solution of Fe(III) to the stirred water glass/CTAB/H_2_O synthesis mixture was delayed by 2 h to allow some extent of SiO_2_ condensation prior to Fe(III) incorporation. This produced a 16 wt% Fe-MCM-41 material with reduction features shown in Figure SI 7 (see Figure SI 8 for the XRD pattern). The first reduction peak corresponds immediately to the Fe_2_O_3_ → Fe_3_O_4_ reduction, while the shoulder at ~503 °C may be assigned to the Fe_3_O_4_ → FeO, and the peak at ~669 °C can be assigned to the FeO → Fe^0^ reduction step. A similar observation was reported by Nesterenko *et al.* [46] on the reducibility of ~4.5 wt% Fe-MCM-41, which showed two peaks at 420 and 600 °C, as well as a shoulder at ~500 °C. The corresponding XRD pattern of this material suggests that the Fe species is either amorphous or too small to be detected by XRD. On the other hand, the use of calcined Si-MCM-41 as a SiO_2_ source and a water solution of Fe(III) in the hydrothermal synthesis of 16 wt% 

**Figure 11:**
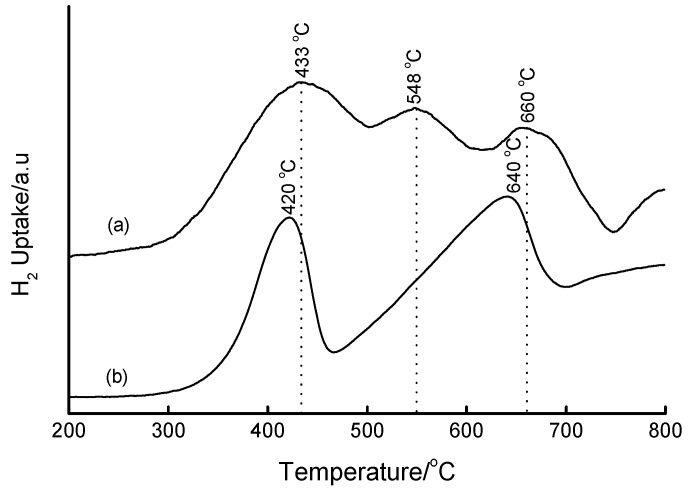
TPR profiles of (a) 16 wt% Fe-MCM-41 prepared hydrothermally from Fe^3+^(aq) and Si-MCM-41 as a SiO_2_ source followed by calcination at 560 °C for 6 h, and (b) bulk Fe_2_O_3_.

Fe-MCM-41 produced reduction features shown in Figure 11. Instead of the commonly accepted two-stage reduction of hematite, 3 Fe_2_O_3_ → 2 Fe_3_O_4_ → 6 Fe, Figure 11 shows the existence of a three-stage reduction process with the three reduction peaks occurring in the temperature range matching the complete reduction of Fe_2_O_3_ to Fe^0^, *i.e.,* at 433, 548 and 660 °C. By analogy with bulk Fe_2_O_3_, the peak at 433 °C is associated with the Fe_2_O_3_ → Fe_3_O_4_ reduction process, while the one at 660 °C is associated with the Fe_3_O_4_ → Fe reduction. The observed peaks are shifted to higher reduction temperatures due to the stabilization of iron oxides by the support. The third reduction peak at 548 °C suggests reduction of Fe_3_O_4_ to the rarely-observed metastable intermediate, FeO, that is known to take place in the temperature range 450–570 °C [44,45,47,48,49,50]. However, it is more likely that the peaks arise from the reduction of the Fe in the structure of the SiO_2_ matrix, *i.e.,* Fe in tetrahedral sites. This interpretation is based on ESR observations that some fraction of Fe exists in both tetrahedral and octahedral sites in these materials.

**Figure 12:**
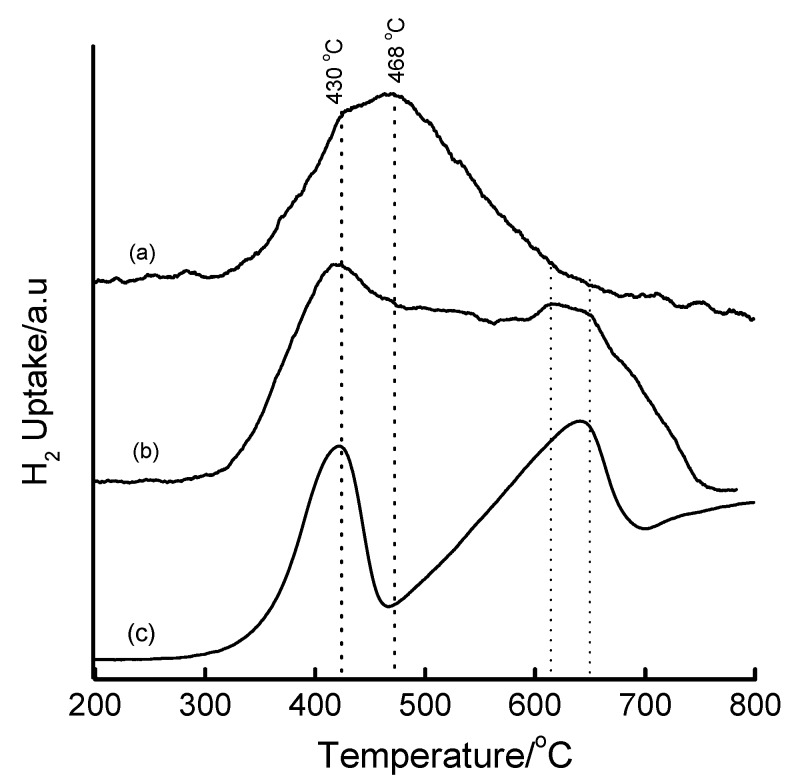
TPR profiles of Fe-MCM-41 prepared via NaOH precipitation of Fe(III) prior to mixing with the Si-MCM-41 synthesis gel and carrying out hydrothermal synthesis at 100 °C for 2 days: (a) 5 wt% Fe, (b) 10 wt% Fe, and (c) bulk Fe_2_O_3_.

Representative Fe-MCM-41 materials prepared by the NaOH precipitate route show reduction patterns depicted in Figure 12. A broad unsymmetrical reduction peak centred at 468 °C is observed for the 5 wt% Fe-MCM-41. This peak corresponds to the reduction step Fe_2_O_3_ → Fe_3_O_4_, and the shift to higher temperatures relative to bulk Fe_2_O_3_ suggests the stabilization of Fe(III)/Fe(II) by the silica matrix. Although the existence of this single reduction peak with a shoulder at 430 °C may suggest only reduction to Fe_3_O_4_ and that the Fe species in this material is difficult to reduce to the metallic state, we cannot rule out the possibility of a simultaneous direct reduction of Fe_2_O_3_ to Fe^0^. Such an observation has been recently reported by Szegedi *et al.* [51,52], who observed a broad peak centred at 670 K (397 °C) for Fe-MCM-41 materials prepared by direct synthesis. Upon increasing the iron content to 10 wt%, the material showed two extra reduction peaks. The first peak appears at ~427 °C (attributable to Fe_2_O_3_ → Fe_3_O_4_) and the second at ~644 °C (attributable to Fe_3_O_4_ → Fe^0^) and suggests the presence of iron both inside and outside the silica framework.

**Figure 13:**
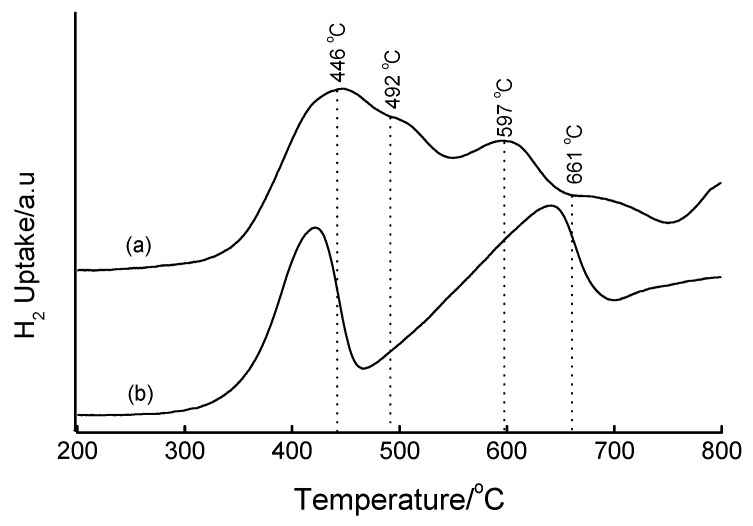
TPR profiles of (a) 16 wt% Fe-MCM-41 prepared by delayed addition of Fe(OH)_3_ to the synthesis gel followed by hydrothermal treatment at 100 °C for 2 days, and (b) bulk Fe_2_O_3_.

**Figure 14:**
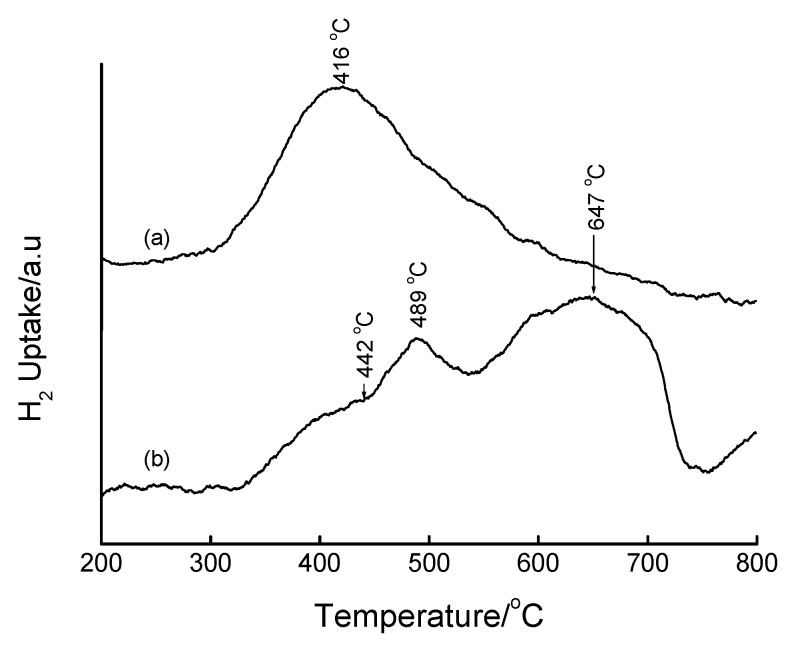
TPR profiles of 16 wt% Fe-MCM-41 prepared at 100 °C for 2 days via the OH^-^ precipitate route: (a) Na_2_CO_3_ and (b) (HOCH_2_CH_2_)_3_N as precipitant.

Prolonging the hydrothermal synthesis of 16 wt% Fe-MCM-41 produces a material that reduces predominantly to Fe_3_O_4_ and negligibly to Fe^0^ (Figure SI 9). It can be speculated at this stage that prolonged synthesis times increases the chances of incorporating Fe in the structure, hence rendering it difficult to reduce. Importantly, materials prepared by the base precipitate method are less reducible than their counterparts prepared by the IWI method (see below). A 2 h delay of the addition of Fe(OH)_3_ to the synthesis gel led to a 16 wt% Fe-MCM-41 material with reduction features at 446, 492 and 597 °C, signifying the existence of the reduction species Fe_2_O_3_ → Fe_3_O_4_ → FeO → Fe^0^, as well as an extra peak beyond 661 °C that can be assigned to the Fe silicate species (Figure 13). Since this material achieves reduction to metallic Fe at lower temperatures compared to bulk Fe_2_O_3_, an improved dispersion of the Fe species can be inferred. As was observed in Figure SI4, the nature of the precipitant affects the crystallinity of the Fe oxide phase and consequently its reducibility (see Figure 14). The use of TEA as precipitant produced 16 wt% Fe-MCM-41 with superior reducibility (with three reduction steps) compared to that obtained when Na_2_CO_3_ is used (only one reduction peak). This is partially supported by the XRD patterns *in Figure SI4,* which suggest a less crystalline iron oxide phase in the Na_2_CO_3_-derived material.

Data for the hydrothermally-fabricated Fe-containing mesoporous silica samples showing the reduction patterns can be summarized in tabular form (Table 3).

**Table 3:** Novel Fe-MCM-41 materials showing a three-step reduction pattern and their synthesis parameters (all calcined at 560 °C for 6 h).

Method	Loading/wt%	LT peak/°C	IT peak/°C	HT peak/°C
Bulk Fe_2_O_3_	-	420	-	640
HDT/MSiO_2_	16	433	548	660
IWI/2^o^ MSiO_2_	5	387	497	661

HDT = hydrothermal, MSiO_2_ = Si-MCM-41, 2^o^ = secondary, LT = low temperature, IT = intermediate temperature, HT = high temperature.

This table shows the similarity in reduction behaviour between hydrothermal Fe-*sec*-Si-MCM-41 and the material obtained by IWI, showing the peak in the temperature range of ~450–570 °C. The ease of reducibility of the Fe species on variants of *sec*-Si-MCM-41 can be rationalized as follows: *sec*-Si-MCM-41 has a restructured morphology because it is synthesized from an already ordered precursor, which results in thickened pore walls which resist penetration by Fe to avoid the formation of irreducible iron silicates through strong metal-support interactions. This resistance allows the high dispersion of Fe species on the restructured mesoporous SiO_2_ support, thus leading to improved reducibility properties due to the presence of Fe_3_O_4_ crystallites.

**Figure 15:**
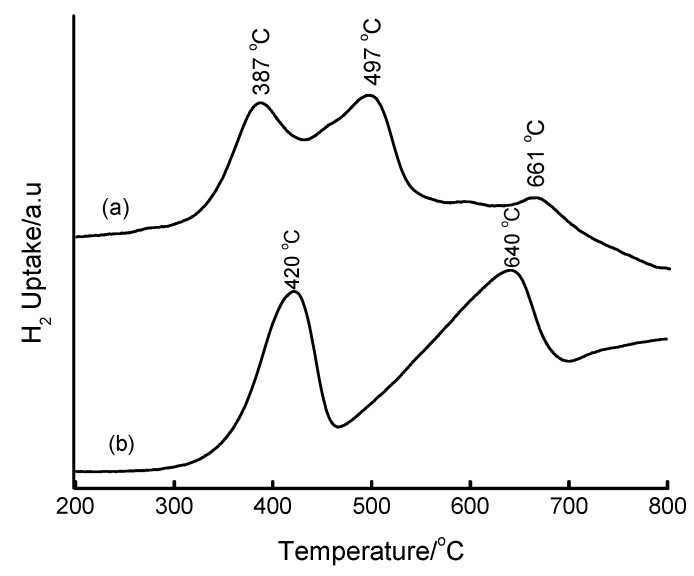
TPR profiles of (a) 5 wt% Fe-MCM-41 prepared by IWI method using *sec*-Si-MCM-41 as a support, calcined at 560 °C for 6 h, (b) bulk Fe_2_O_3_.

**Figure 16:**
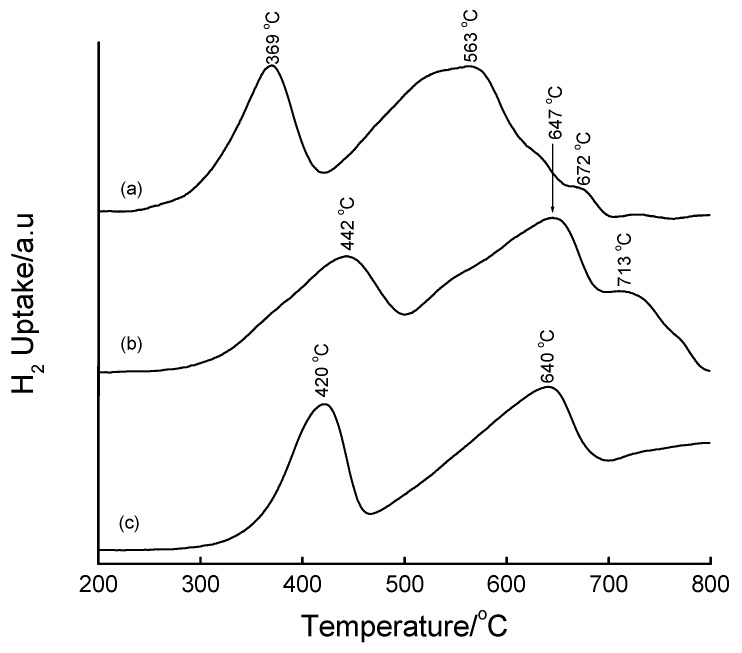
TPR profiles of 16 wt% Fe-MCM-41 prepared by IWI: (a) calcined at 450 °C for 12 h, (b) calcined at 560 °C for 6 h, and (c) bulk Fe_2_O_3_ reference.

TPR profiles of the incipient wetness impregnated samples are shown in Figure 15 and Figure 16. The TPR profiles of the Fe-containing samples show that iron is present as Fe_2_O_3_ before reduction. The most interesting reduction pattern is observed when *sec*-Si-MCM-41 is used as a support, wherein three reduction peaks appear at 387, 497 and 661 °C. On the primary Si-MCM-41 (Figure 16), two reduction peaks and a shoulder are observed within a temperature range coinciding with the reduction process Fe_2_O_3_ → Fe_3_O_4_ → Fe^0^ (*cf*. bulk Fe_2_O_3_). Both the second reduction peak and the shoulder correspond to the reduction of Fe_3_O_4_ to Fe^0^, with the shoulder representing Fe in strong interaction with the support. The symmetry of the main peak (2^nd^) is compatible with that of the corresponding peak in the TPR profile of Fe_2_O_3_ and therefore assigned to the Fe_3_O_4_ → Fe^0^ reduction step. Another notable feature in Figure 16 is the shift of the reduction peaks to higher temperature upon increasing the calcination temperature from 450 °C to 560 °C, a phenomenon that has been attributed to enhanced metal-support interactions [28,29,53]. It can be concluded that calcination at 450 °C produces highly dispersed Fe species on 1^o^ Si-MCM-41 and that higher calcination temperatures induces stronger metal-support interactions which renders the reduction of the Fe species difficult.

## 4. Conclusions

High Fe loadings with retention of mesoporosity and long-range order in the silica matrix can be achieved in one-pot syntheses by using Si-MCM-41 as a SiO_2_ source instead of water glass, and also by delaying the addition of the metal precursor (either aqueous or hydroxide) to the synthesis gel by ~2 h. However, the latter method involving the metal hydroxide is limited by uncontrolled dispersion of the metal precursor and consequent clustering, *i.e.,* most of the Fe exists as a separate phase clustering over the surface of Si-MCM-41.

The Fe-MCM-41 materials prepared by the base precipitate route over 2 days show various reducibility patterns, generally ranging from a broad 1-peak to 3-peak profile. The multi-step reduction pattern was also observed for the materials prepared by delayed (2 h) addition of Fe(OH)_3_ to the synthesis gel prior to hydrothermal treatment, which showed the reduction of the Fe-silicate species in addition to the 3-peak process Fe_2_O_3_ → Fe_3_O_4_ → FeO → Fe^0^. A notable 3-peak reduction pattern was also observed for the16 wt% Fe-MCM-41 material obtained via the TEA precipitate route, a feature that correlated with the metal oxide peaks in the XRD pattern.

The Fe-MCM-41 materials involving *sec*-Si-MCM-41, either through hydrothermal synthesis or through IWI, exhibit maximal retention of mesoporous structural order, as well as enhanced reducibility taking place through the well-defined 3-step process. Since primary Si-MCM-41 collapses under hydrothermal conditions (due to its thin and amorphous pore walls), the ability of *sec*-Si-MCM-41 to survive under hydrothermal conditions makes it a potential support for processes such as the FTS, which produces vast amounts of water during the reaction. 

In summary, a novel synthesis method has been identified for the synthesis of highly-loaded Fe-MCM-41 with preservation of the mesoporous structure and long-range order. The method involves secondary synthesis in the presence of and Fe precursor. In addition, the resulting materials show interesting reducibility patterns, making them good candidates as heterogeneous catalysts and for the FTS. In addition to the conventional interpretation of the three-step reduction events as involving wustite (FeO), we also propose the third reduction peak arises from the reduction of the Fe in the structure of the SiO_2_ matrix, *i.e.,* Fe in tetrahedral sites.

## Supplementary Materials

Supplementary materials can be downloaded online at http://www.mdpi.com/1996-1944/2/4/2337/s1/.
