# Supplementary material for: Highly Loaded Fe-MCM-41 Materials: Synthesis and Reducibility Studies
Source: Materials (Basel). 2009 Dec 15;2(4):2337–59. doi: 10.3390/ma2042337 (PMC5513583; doi:10.3390/ma2042337)
Supplement: Supplementary file 1 [file materials-02-02337-s001.pdf]

Supplementary Materials

Highly Loaded Fe MCM-41 Materials: Synthesis and Reducibility Studies

Malose P. Mokhonoana [1] and Neil J. Coville [2,*]

Figure SI 1. Variation of the lattice parameter (A) and BET surface area (B) with Fe content for the Fe-MCM-41 materials prepared in 1 M HNO_3 at 100 °C for 48 h. All samples were calcined at 560 °C for 6 h.

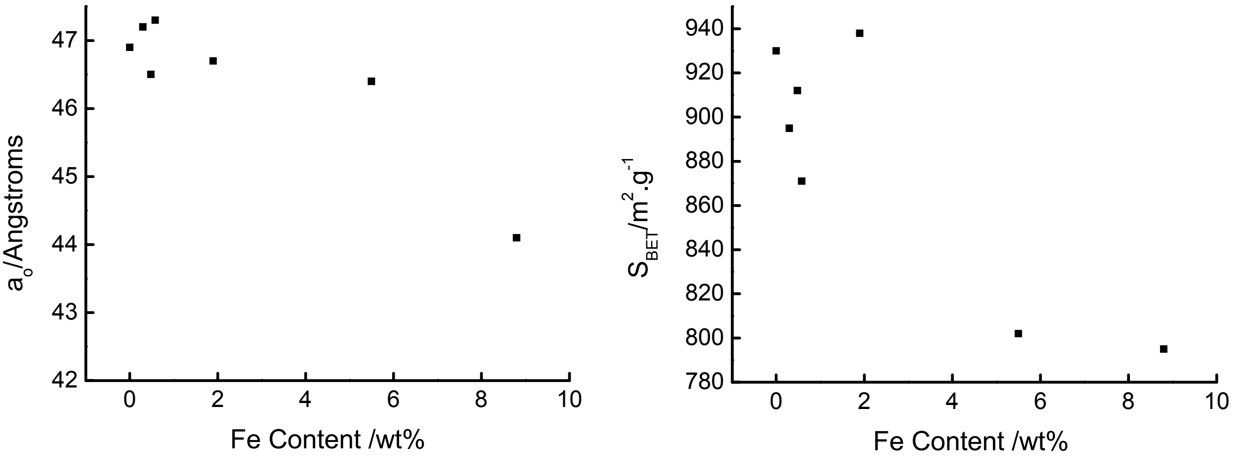

(A) (B)

Figure SI 2. The full-range XRD plot of the XRD pattern of the 16 wt% Fe-MCM-41 material synthesized hydrothermally using calcined Si-MCM-41 as a SiO_2 source.

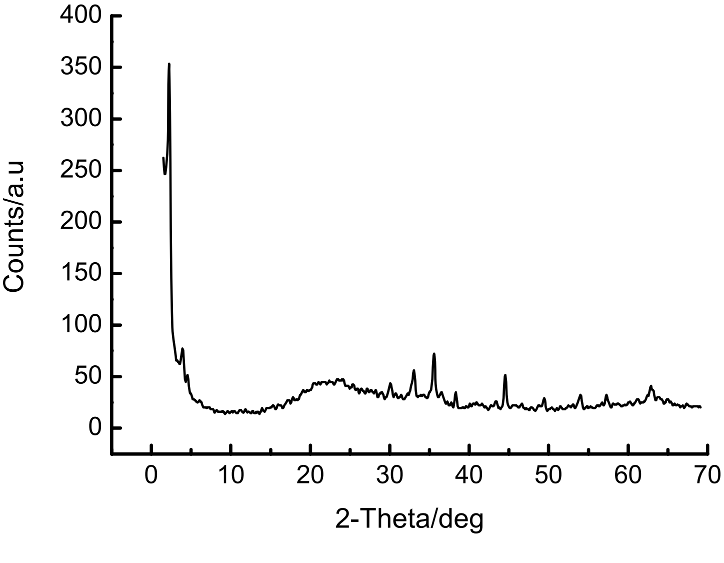

Figure SI 3. HRTEM micrograph of 3 wt% Fe-MCM-41 prepared at 80 °C for 6 h using water glass as SiO_2 source. The material was then calcined at 500 °C for 12 h.

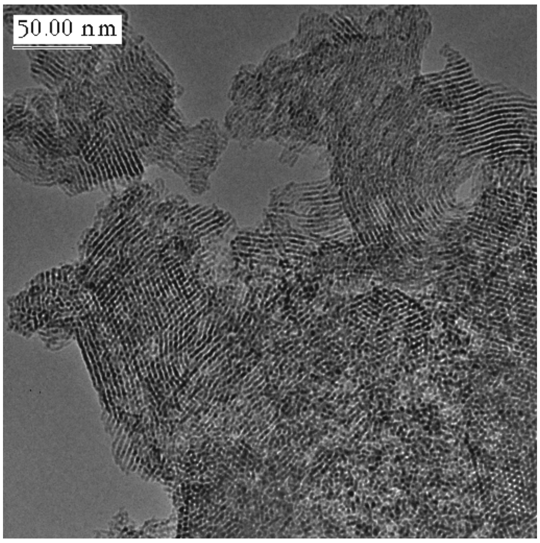

Figure SI 4 shows that the nature of the precipitating agent has an influence on the structural and textural properties of the 16 wt% Fe-MCM-41. The XRD patterns in *Figure SI 4* show retention of the mesostructure of MCM-41, together with its long-range order of hexagonal channels. In addition, Fe_2O_3 peaks appear in the high-angle region of the XRD patterns, which are more intense for NaOH and triethanolamine (TEA) than for Na_2CO_3. Notably, the metal oxide peaks for the TEA-based material between 20 and 40 °2θ are more intense than the NaOH-based counterpart. These peaks may be related to the reducibility of these materials (see TPR section).

Figure SI 4. XRD patterns of 16 wt% Fe-MCM-41 synthesized hydrothermally using the base precipitate route at 100 °C for 2 days. Materials were calcined at 560 °C for 6 h.

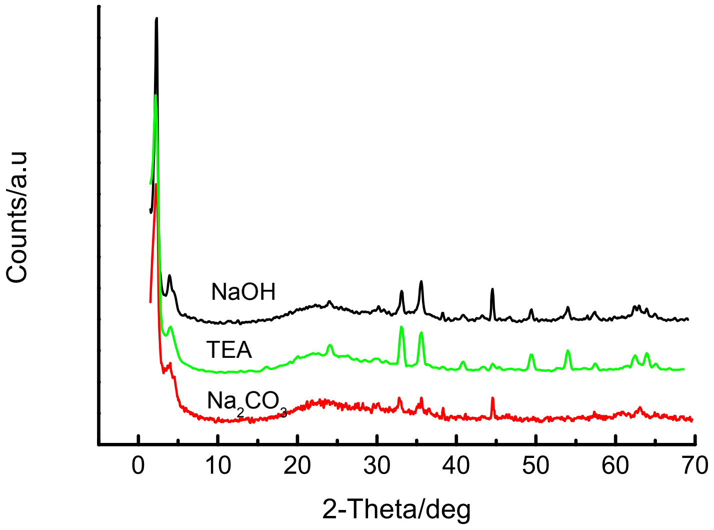

Figure SI 5. Variation of the lattice parameter (a_o) as a function of Fe loading for Fe-MCM-41 prepared by incipient wetness impregnation of Si-MCM-41 prepared at 100 °C for 2 days.

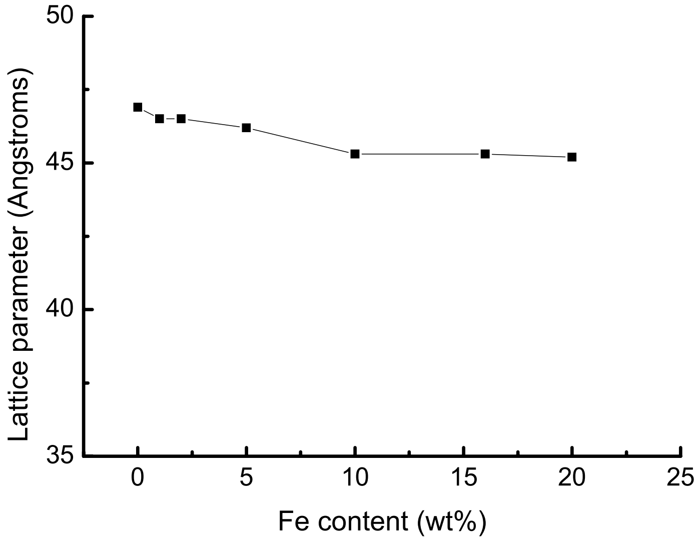

Figure SI 6. XRD patterns of Fe-MCM-41 prepared by IWI compared with that of bulk Fe_2O_3: (a) 16 wt% Fe-MCM-41, (b) 50 wt% Fe-MCM-41 and (c) bulk Fe_2O_3 (Merck)

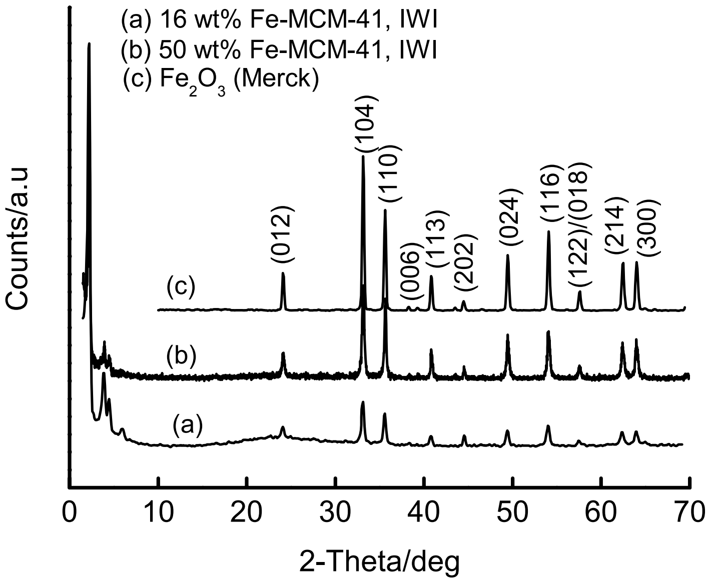

Figure SI 7. TPR profiles of (a) 16 wt% Fe-MCM-41 prepared by adding Fe^{3+}(aq) to the Si-MCM-41 synthesis gel 2 h after mixing (but prior to hydrothermal treatment and calcination at 560 °C for 6 h) and (b) bulk Fe_2O_3.

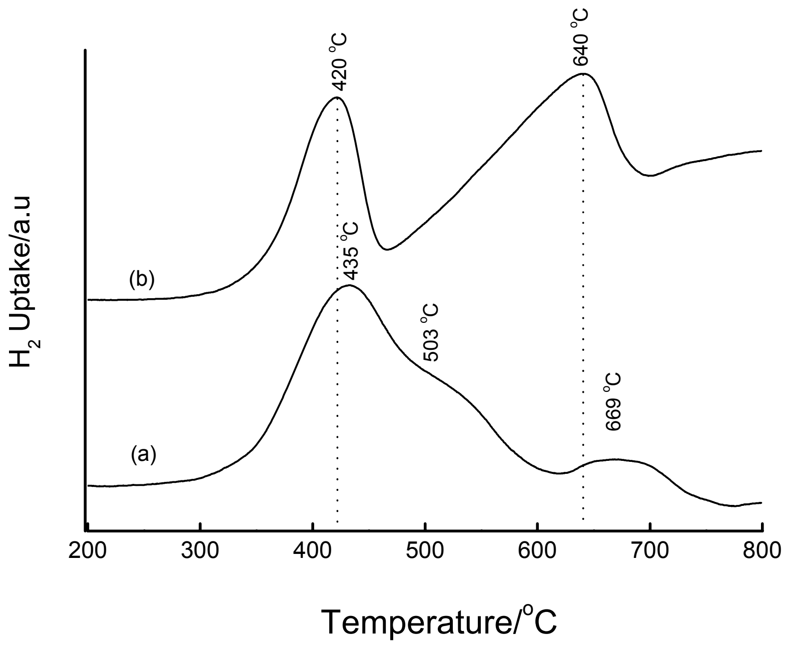

Delaying the addition of the water solution of Fe(III) to the mixed water glass/CTAB/H_2O gel by 2 h produced a 16 wt% Fe-MCM-41 with some degree of hexagonal order as shown below.

Figure SI 8. Low-angle (*left*) and high-angle (*right*) XRD patterns of 16 wt% Fe-MCM-41: A water solution of Fe(III) added 2 h after mixing the water glass/CTAB/H_2O gel, and the synthesis carried out at 100 °C for 6 h.

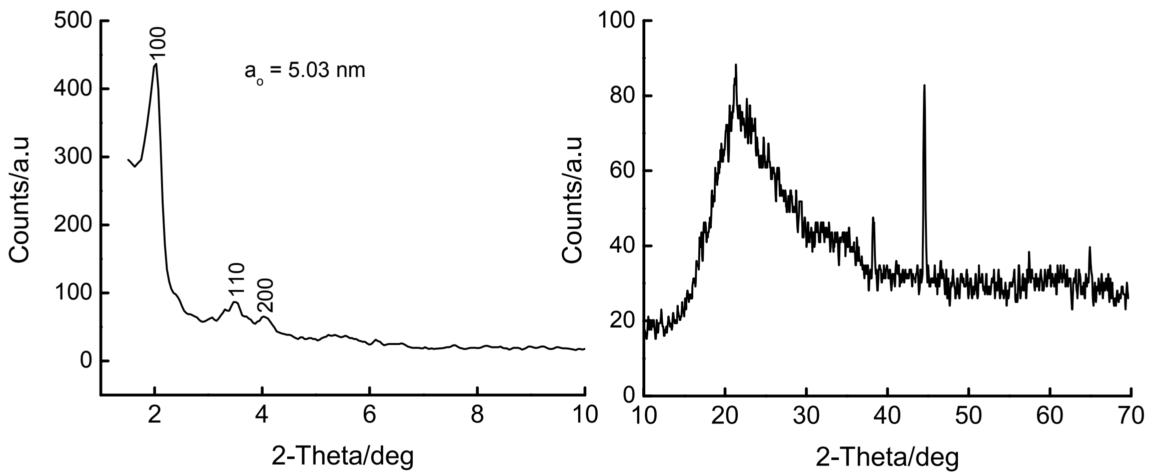

Figure SI 9. TPR profiles of 16 wt% Fe-MCM-41 prepared via the OH^- route at 100 °C for 5 days: (a) calcined at 450 °C for 12 h, and (b) calcined at 560 °C for 6 h.

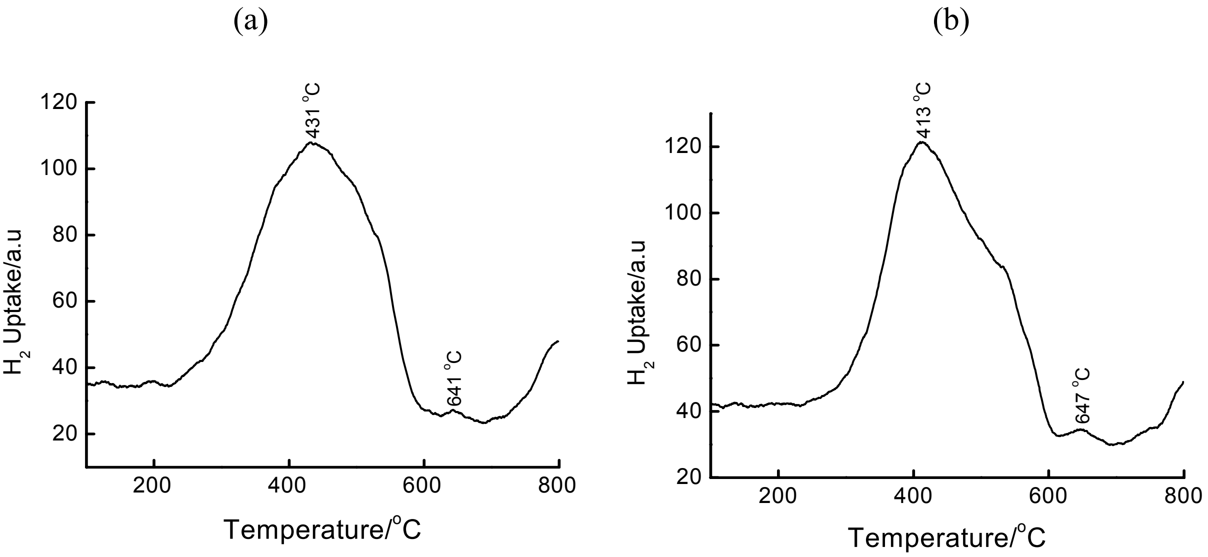

Table SI 1. A summary of the structural and textural data of the Fe-MCM-41 materials prepared hydrothermally via the OH^- precipitate route.

Wt% Fe	a_0/Å[a]	$S_{BET}/m^2.g^{-1}$ sample[b]	$S_{BET}/m^2.g^{-1}$ support[c]
0	47.0	930	930
5	45.3	879	926
10	43.8	691	768
16	44.4	546	650
20	46.2	429	536

[a] Error bar is ± 0.6, [b] Surface area per gram of Fe-MCM-41, [c] Surface area per gram of Si-MCM-41, *i.e.,* the mass of Fe-MCM-41 corrected for the mass of Fe in the sample to leave only the SiO_2 component.
